# Expression of progenitor cell/immature neuron markers does not present definitive evidence for adult neurogenesis

**DOI:** 10.1186/s13041-019-0522-8

**Published:** 2019-12-10

**Authors:** Hideo Hagihara, Tomoyuki Murano, Koji Ohira, Miki Miwa, Katsuki Nakamura, Tsuyoshi Miyakawa

**Affiliations:** 1 Division of Systems Medical Science, Institute for Comprehensive Medical Science, Fujita Health University, Toyoake, Aichi 470-1192 Japan; 2 Laboratory of Nutritional Brain Science, Department of Food Science and Nutrition, Mukogawa Women’s University, Nishinomiya, Hyogo 663-8558 Japan; 3 Cognitive Neuroscience Section, Primate Research Institute, Kyoto University, Inuyama, Aichi 484-8506 Japan

**Keywords:** Hippocampus, Dentate gyrus, Granule cells, Dematuration, Adult neurogenesis

## Abstract

It is agreed upon that adult hippocampal neurogenesis (AHN) occurs in the dentate gyrus (DG) in rodents. However, the existence of AHN in humans, particularly in elderly individuals, remains to be determined. Recently, several studies reported that neural progenitor cells, neuroblasts, and immature neurons were detected in the hippocampus of elderly humans, based on the expressions of putative markers for these cells, claiming that this provides evidence of the persistence of AHN in humans. Herein, we briefly overview the phenomenon that we call “dematuration,” in which mature neurons dedifferentiate to a pseudo-immature status and re-express the molecular markers of neural progenitor cells and immature neurons. Various conditions can easily induce dematuration, such as inflammation and hyper-excitation of neurons, and therefore, the markers for neural progenitor cells and immature neurons may not necessarily serve as markers for AHN. Thus, the aforementioned studies have not presented definitive evidence for the persistence of hippocampal neurogenesis throughout adult life in humans, and we would like to emphasize that those markers should be used cautiously when presented as evidence for AHN. Increasing AHN has been considered as a therapeutic target for Alzheimer’s disease (AD); however, given that immature neuronal markers can be re-expressed in mature adult neurons, independent of AHN, in various disease conditions including AD, strategies to increase the expression of these markers in the DG may be ineffective or may worsen the symptoms of such diseases.

## Introduction

**Fig. 1:**
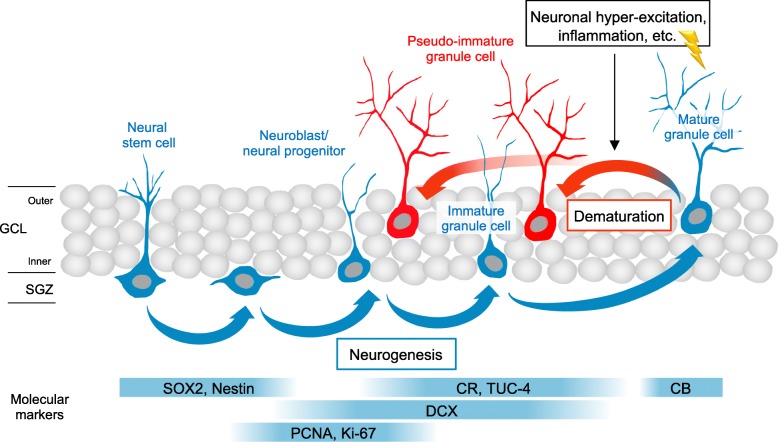
Immature neuronal markers expressed during dematuration and neurogenesis. Dematuration of mature neurons can be induced by several factors, such as neural hyper-excitation and inflammation. Consequently, immature neuronal markers can be re-expressed in pre-existing mature neurons, independent of adult neurogenesis. GCL, granule cell layer. SGZ, subgranular zone

There is an agreement that new neurons are generated in the dentate gyrus (DG) of hippocampus in rodents and non-human primates during adulthood, but it remains to be determined whether the adult hippocampal neurogenesis (AHN) commonly occurs in humans throughout aging. Recently, Moreno-Jiménez and colleagues reported that there are a number of neurons in the human DG that express immature neuron markers, such as doublecortin (DCX) and calretinin (CR), which are believed to be “reliable” neurogenic markers (See Additional file 1: Table S1), claiming that this provides evidence for the persistence of AHN in aged humans [1]. Other studies by Boldrini et al. [2] and Tobin et al. [3] that used similar markers also arrived at the same conclusions. Moreno-Jiménez et al. reported decreased expression of immature neuronal markers in patients with Alzheimer’s disease (AD) compared with neurologically healthy subjects [1], which was not replicated by Tobin et al. [3]. However, these immature neuronal markers can be re-expressed in pre-existing mature neurons by neural excitation and drug administration, and their expression can be independent of adult neurogenesis (Fig. 1). Therefore, data shown by the above-mentioned studies may not serve as definitive evidence of the existence of human adult neurogenesis. Herein, we briefly review the previously published literature that have demonstrated that immature neuronal markers, such as DCX and CR, can be re-expressed in pre-existing mature neurons via a process that we refer to as dematuration. We note the possibility that molecular expression pattern indicative of immature neurons might occur in the DG of patients with AD by demonstrating the similarity between the molecular expression patterns observed in the DG of patients with AD and that of mice with an “immature dentate gyrus” phenotype.

### Re-expression of cell cycle/progenitor markers in mature neurons by cell cycle re-entry

A series of studies have shown ectopic expression of stem cell / cell cycle markers, such as Sox2, PCNA, and Ki-67, in mature postmitotic neurons, including granule cells (GCs), in the hippocampal DG in the brains of patients with AD and other neurodegenerative disorders [4, 5] (for references, see Additional file 1: Table S2). It has been demonstrated that neuronal death is preceded by re-expression of cell cycle markers in mature neurons and that various insults, including toxic concentrations of amyloid-βand hyper-excitation of neurons induced by kainic acid, can lead to the expression of these markers [4]. This cell cycle re-entry of postmitotic neurons is postulated as an additional or alternative mechanism contributing to the symptoms of AD [6]. Therefore, the expression of these cell cycle markers may not necessarily indicate AHN. Although cells expressing these markers were seen in the subgranular zone (a well-known neurogenic region) and the middle and outer parts of the granule cell layer (GCL; where mature granule cells are typically located) [2, 3], a significant proportion of these expressions, albeit not all, especially within the GCL, could be contributed by the re-entry of mature GCs into the cell cycle, rather than due to AHN.

### Supporting evidence for dematuration of mature neurons

There are several lines of evidence supporting the existence of dematuration in particular types of neurons [7–13]. Our study, along with other studies, has demonstrated that the maturation of GCs in the DG is a dynamic and reversible process, involving changes in their molecular expression patterns and electrophysical properties [7, 8] (Fig. 1; Additional file 1: Table S2). Chronic treatment with the antidepressant, fluoxetine (FLX), causes the reversal of mature adult GCs back to a pseudo-immature status [7]. Mice were injected with bromodeoxyuridine (BrdU), which gets incorporated into the newly synthesized DNA strand of proliferating cells and can be used as a cytogenetic marker, at neonatal period (postnatal days 1–3), and thereafter, treated with FLX or vehicle at adulthood (at 9 weeks of age) for 4–5 weeks. It was found that in the adult DG, BrdU-positive (BrdU^+^) cells, i.e., putative mature GCs, expressed calbindin (CB), a marker of mature GCs, in the vehicle-treated control mice. However, many BrdU^+^ cells lacked CB expression in FLX-treated animals, indicating that the mature neurons lost CB expression as a result of FLX treatment [7]. Newly generated immature GCs in adults are located in the inner part of the GCL or the subgranular zone, as assessed by BrdU labeling. However, the reduction in CB and the overexpression of DCX and CR, which are also immature GC markers, can also be found in the middle or outer layer of the GCL in FLX-treated animals [7], supporting the idea that “dematuration,” the process by which mature GCs return to a pseudo-immature state, can be induced in GCs by FLX treatment. CB, along with more than 380 mature marker genes and 150 immature marker genes, were down or up-regulated in the DG of the mice that received chronic treatment with FLX, which cannot be explained solely by an increase in AHN [8].

Dematuration of GCs can also result from epileptic seizures induced by pilocarpine [13] and electroconvulsive shocks [14] in rodents, as assessed by CB expression, genome-wide gene expression patterns, and/or the electrophysiological properties of GCs. Mice with mutant synaptosomal-associated protein 25 (SNAP25) exhibit epileptic seizures occasionally and have the so-called “immature dentate gyrus” phenotype, which can be treated by preventing epileptic seizures via the administration of an anti-epileptic drug [15]. Decreased expression of CB has also been observed in the DG of patients with epilepsy [16, 17], and the expression of CB is also decreased in the DG of AD mouse models and AD patients [18, 19]. Considering that epileptic seizures have been observed in patients with AD [20], and seizure frequency is negatively correlated with CB levels in the DG of AD model mice [18], this decreased expression of CB in the DG may be a result of the epileptic seizures. Gene expression signatures of neuronal hyper-excitation and immaturity have been found post-mortem in the brains of patients with AD, schizophrenia, and amyotrophic lateral sclerosis [21]. These findings indicate that hyper-excitation of neurons can cause dematuration in the brains of rodents and, probably humans, including AD patients.

### AHN and dematuration are separate phenomena

AHN and dematuration are separate phenomena, since dematuration in the DG is accompanied by increased, decreased, or unchanged AHN. Increased AHN is a well-established and intensely investigated phenomenon, including in mice that were chronically treated with FLX [22, 23]. Increased adult neurogenesis was seen in a few different kinds of mice with an “immature dentate gyrus” phenotype [11, 14, 24]. On the contrary, in SNAP25 mutant mice with an “immature dentate gyrus” phenotype, the AHN, as assessed by BrdU labeling, was almost absent [15]. The relative position of DCX^+^ cells shifted from the subgranular zone to the middle or outer GCL in these mutant mice. However, it is unlikely that DCX expression in the middle or outer GCL is due to AHN, because BrdU staining was mostly restricted to the subgranular zone in these mutant mice, as found in the wild-type mice.

In the DG of common marmosets, the expression levels of immature GC markers (e.g., DCX and CR) were increased without any increase in adult neurogenesis in response to chronic antidepressant treatment, as assessed by BrdU staining, which is a more robust cytogenesis marker [10]. It should be noted that a number of DCX^+^ cells were observed in the middle and outer GCL, while BrdU^+^ cells were only observed in or close to the subgranular zone, exemplifying that adult neurogenesis and dematuration were separate phenomena, and demonstrating that the expression of immature GC markers does not necessarily serve as evidence for AHN in non-human primates. AHN is thought to occur in or close to the subgranular zone, which was also seen through BrdU staining of the DGs of non-human primates [10]. Figure 2 in the paper by Tobin et al. [3] shows that DCX^+^ cells are located not only around the subgranular zone, but also in the middle and outer parts of the GCL, where mature GCs are typically located, in elderly humans and AD patients, raising the possibility that at least some of the DCX^+^ cells are GCs that underwent dematuration, rather than being newly generated. Identifying the positions of cells that express immature GC markers within the GCL, either near to or far from the subgranular zone, might be one way to discriminate whether those cells are derived from neurogenesis or dematuration. Considering that both mature astrocytes and postmitotic neurons that express DCX have been observed in the cortex [25, 26], the possibility of the existence of cell types, other than GCs that have undergone dematuration, that express immature GC markers should also be taken into account. In addition, if neurogenic markers that are expressed independent of dematuration are identified in future studies, they could provide a useful means to discriminate these two phenomena.

Ectopic expression of immature neuronal markers can be found in the middle and outer GCL of patients with epilepsy, which presents the possibility that these neurons may also be formed by adult neurogenesis [27]. If this mechanism also existed in FLX-treated marmosets and SNAP25 mutant mice, upon BrdU incorporation assay, DCX^+^ or CR^+^ GCs located in the middle and outer GCL should have been labeled with BrdU, which should have been incorporated in the process of adult neurogenesis. However, the positions of the DCX^+^ or CR^+^ cells and BrdU^+^ cells differed from each other within the GCL in FLX-treated marmosets and SNAP25 mutant mice; a number of DCX^+^ or CR^+^ cells were located in the middle and outer regions of the GCL, whereas almost all of the BrdU^+^ cells were confined to the subgranular layer in these animals. Thus, the expression of immature neuronal markers in the middle and outer GCL may not be attributed to adult neurogenesis, but rather to the dematuration of mature neurons.

Re-expression of cell cycle and immature markers in mature GC neurons does not completely disprove the existence of human AHN. Strong evidence for AHN in humans has been provided by a birth-dating study of dividing precursor cells using ^14^C incorporation into synthesized DNA, which did not rely on the detection of molecular markers [28]. It has been suggested, however, that the extent of AHN may be overestimated in immunohistochemical studies of immature marker expression in comparison to ^14^C birth-dating data [29]. This potential overestimation may be due to that GCs that underwent dematuration were also considered as immature neurons derived from AHN in immunohistochemical studies.

### Dematuration of mature neurons as a hallmark of AD and aging

If cells dedifferentiate in diseased or damaged conditions, such as in AD, the proportion of cells expressing immature neuronal markers should be greater in the brains of patients with AD. Indeed, many previous reports show increased expression of immature GC markers in the brains of patients with AD [30–32] and other neurodegenerative disorders [33], whereas Moreno-Jiménez et al. reported decreased expression of immature neuronal markers in the DG of patients with AD. Also, Tobin et al. found no statistically significant decrease in the number of cells expressing immature GC markers in patients with AD as compared to control individuals [3]. Therefore, one might argue that it is inconsistent with the hypothesis that dematuration or cell cycle re-entry is increased in patients with AD. In fact, however, in Figure 2 and Figure 4 of the report by Tobin et al., patients with AD showed the highest level of DCX^+^ or DCX^+^/PCNA^+^ cell numbers [3]. In addition, considering that 1) those markers are overexpressed in many brain regions other than the DG in patients with AD, and 2) neuronal loss in the hippocampus of patients with AD happens mostly in the CA1 area and not much in the DG [34–36], these cell cycle / immature neuronal markers need not be overexpressed exclusively in the DG of patients with AD (the most affected brain region may differ among individual patients). Moreover, a decrease of DCX expression does not necessarily mean reduced dematuration in these patients. As mentioned above, SNAP25 mutant mice exhibit severe dematuration phenotypes in the DG with regard to CB expression, genome-wide gene expression patterns, and electrophysiological properties, whereas DCX expression is significantly decreased in these mice [15]. Thus, the potential increase of dematuration in patients with AD may not be inconsistent with decreased DCX expression.

Further, we would like to mention that dematuration can occur even in seemingly normal neurons. Differentiated neurons can re-express cell cycle markers by neuronal hyper-excitation [37, 38]. It is easy to imagine that such neuronal hyper-excitation can occur in apparently healthy aged individuals. Interestingly, there is a group of putative marker genes for immature neurons that show upregulation during brain development, and tend to exhibit peak expression at approximately 26 years of age and gradually decrease with aging [39]. This suggests that a dematuration-like phenomenon, demonstrated by gene expression patterns, may occur in healthy, aging individuals.

## Conclusions

Given that cell cycle and immature GC markers can be re-expressed in mature adult neurons, independent of AHN in rodents and primates, it is quite possible that at least a part of the expression of those markers in the human DG may not be due to AHN, but due to the cell cycle re-entry /dematuration of old mature neurons. Re-expression of cell cycle and immature markers in mature GC neurons does not completely disprove the existence of human AHN. However, it contradicts the claim that their enhanced expression levels serve as the definitive evidence of its existence and strongly indicates the overestimation of the amount of AHN, if any. Thus, we believe it is premature to conclude that AHN can truly occur in humans to the extent reported by Moreno-Jiménez et al. [1], Boldrini et al. [2], and Tobin et al. [3]. Moreover, we would like to draw attention to the possibility that the re-expression of the markers for cell cycle/immature neurons may contribute to the pathophysiology of the disease and that the strategy to simply increase the expression of these markers may be ineffective or may rather worsen the symptoms.

## Supplementary information

**Additional file 1 Table S1.** Molecular evidence for the adult hippocampal neurogenesis in humans. **Table S2.** Literature showing evidence of the expression of stem cell/cell cycle/immature neuron markers in mature neurons.

## Data Availability

Not applicable.

## Abbreviations

AD: Alzheimer’s disease
AHN: Adult hippocampal neurogenesis
BrdU: Bromodeoxyuridine
CA1: Cornus ammonis 1
CB: Calbindin
CR: Calretinin
DCX: Doublecortin
DG: Dentate gyrus
FLX: Fluoxetine
GCL: Granule cell layer
PCNA: Proliferating cell nuclear antigen
SNAP25: Synaptosomal-associated protein 25
Sox2: SRY-box transcription factor 2
